# Editorial: Approaches That Foster a Pro-Regenerative Environment

**DOI:** 10.3389/fbioe.2022.873375

**Published:** 2022-03-09

**Authors:** Carl A. Gregory, Ryang Hwa Lee, Fei Liu, Daniel Alge

**Affiliations:** ^1^ Department of Molecular and Cellular Medicine, Texas A&M University Health Science Center, Bryan, TX, United States; ^2^ College of Engineering, Texas A&M University, College Station, TX, United States

**Keywords:** autotherapy, regeneration, stem cells, immunomodulation, therapy

The term *autotherapy* was originally coined in the early 1900’s, referring primarily to the re-administration of crude preparations of biological fluids (e.g., wound exudates, blood) from diseased patients in an attempt to accelerate inherent healing processes. Reports of its successful implementation were numerous and convincing, especially for infectious diseases, but the therapeutic mechanism of such an intervention was unclear, with hypotheses ranging from modulation of immune responses to purely psychosomatic effects (Duncan, 1914; Duncan, 1917; Greval, 1949). In 2018, Lumelsky and others at the National Institute of Dental and Craniofacial Research (NIDCR) revived the definition of autotherapy, applying it specifically to those approaches that stimulate inherent tissue responses, manipulate stem cell niches, and modulate endogenous tissue microenvironments to enhance tissue healing and regeneration (Lumelsky et al., 2018).

Modern autotherapeutic concepts can be crudely divided into i) strategies that modulate the stem cell niche (e.g., stem cells, modulation or recapitulation of the stem cell niche) ii) those that foster a pro-regenerative environment (immune modulation, anti-inflammatory approaches, angiogenesis, metabolism, microbiome) and (iii) direct lineage reprogramming of stem cells or trans-differentiation of differentiated cells (e.g., epigenetic or genetic modification *in situ* with non-viral agents). As it relates to tissue engineering and regenerative medicine, this could be achieved by the transient modulatory effects of cell therapy, administration of stem-cell derived factors such as extracellular vesicles (EVs), secreted ligands or extracellular matrices (ECMs), or from synthetic small molecules, genetic engineering tools, biomaterials or scaffolds. The entire field of autotherapy is extremely broad, but in this collection, we focus on approaches that foster a pro-regenerative environment through stimulation of stem cells and/or modulation of the immune system.

We begin with Dr Lumelsky’s opinion article (Lumelsky) stressing the importance of local control in the propagation of a pro-regenerative environment. In particular, Dr Lumelsky emphasizes the contribution of the stem cell niche in this process, stressing that its individual components, stem cells, stromal cells, extracellular matrices and soluble mediators play distinct but equally important roles in maintaining regenerative homeostasis. In addition, the contribution of the immune system is discussed, with emphasis on the need for precise modulation of inflammatory and post-inflammatory processes to support tissue regeneration rather than fibrosis. Within this context, the potential of synthetic biomaterials to control cell adhesion characteristics, biomechanical forces, and soluble factor delivery is discussed. In keeping with the emphasis on biomaterials and their potential for immune modulation, Karkanitza et al. reviews our current understanding of immune reactions to biomaterials, and considers how this knowledge might be employed to drive regeneration and reduce the probability of adverse reactions.

One of the major challenges in the translation of experimental therapies to clinical implementation is the selection of appropriate animal models for preclinical testing. In most cases, and especially for musculoskeletal applications, a large animal model is required, mimicking as closely as possible, the load and weight bearing characteristics of the human body. Although largely overlooked, the canine system is an excellent model for skeletal regeneration, with similar anatomy to humans, well-developed surgical techniques, and the capacity to perform gait analysis. Dobson et al. examine the mechanism of canine mesenchymal stem cells (MSCs) during bone formation. Using canine MSCs in mouse recipients, they describe for the first time that MSCs and sub-therapeutic levels of bone morphogenic proteins synergize to drive bone repair. In these studies, the implanted MSCs were essential for rapid repair, but did not persist at the site of injury suggesting that MSCs, when triggered with very low levels of BMP, stimulate the host’s inherent bone repair mechanisms rather than directly differentiate into osteoblasts. This study represents an excellent example of cell-mediated autotherapy and contributes to the rapidly growing number of studies supporting autotherapy as the predominant mechanism of MSC-mediated healing. Han et al. offer an alternative approach for therapeutic enhancement of adipose derived MSCs through *ex vivo* exposure to decellularized adipose tissue ECM. In this work, the authors demonstrate that culturing of MSCs on decellularized adipose tissue ECM enhanced secretion of regenerative and immune-modulatory factors. This process could, in the future, improve the autotherapeutic potential of adult stem cells.

While stem cells have the potential to deliver multiple bioactive factors with the means to adapt to the dynamic healing environment, some autotherapeutic effects can be achieved by simple and direct administration of defined factors. Liebman et al. present an intriguing strategy for the stabilization of labile biological ligands hepatocyte growth factor and fibroblast growth factor 2 by attachment to immunoglobulins. In a porcine model of myocardial ischemia and perfusion, the immunoglobulin-sequestered factors were stable and promoted repair of cardiac tissue. Sankar et al. describe the broad autotherapeutic applications of RAD16, a synthetic peptide with hemostatic properties that also serves as an ECM analog for cell attachment and migration. To address the issue of radiation-induced salivary gland damage during treatment of head and neck cancer, Gilman et al. employed the non-steroidal anti-inflammatory drug indomethacin to inhibit prostaglandin E2-mediated inflammatory processes and promote regeneration of the gland. In a related study, Nam et al. employ hydrogels to deliver Laminin-1 peptides for enhanced autotherapy of salivary glands through stimulation of the salivary stem cell niche and by modulation of macrophages to adopt a pro-regenerative phenotype. Xu et al. demonstrate that simple, naturally occurring substances can be employed as autotherapeutic tools. In their study, a simple preparation of platelet rich plasma stimulated regenerative characteristics of human dental pulp cells, providing some credence to the reports of autotherapy in the early 1900’s.

Fetz et al. and Hymel et al. represent excellent examples of the utilization of synthetic materials to regulate local release of therapeutic factors into the regenerative microenvironment. Fetz et al. employed electrospun polydioxanone to slowly release chloroquine which in turn caused neutrophils to downregulate inflammatory factors and adopt a more resolving, putatively regenerative phenotype. Using a similar approach, Hymel et al. utilized nanofibers to release bioactive lipid into the myogenic microenvironment to enhance myofiber growth and promote regenerative immunological processes.

We hope this diverse collection of studies showcases many aspects of the autotherapy concept and recent efforts in developing innovative approaches to enhance our inherent capacity for tissue healing and regeneration.
